# Breast Cancer Risk and Breast-Cancer-Specific Mortality following Risk-Reducing Salpingo-Oophorectomy in *BRCA* Carriers: A Systematic Review and Meta-Analysis

**DOI:** 10.3390/cancers15051625

**Published:** 2023-03-06

**Authors:** Faiza Gaba, Oleg Blyuss, Alex Tan, Daniel Munblit, Samuel Oxley, Khalid Khan, Rosa Legood, Ranjit Manchanda

**Affiliations:** 1 Institute of Applied Health Sciences, University of Aberdeen, Aberdeen AB24 3FX, UK; 2 Department of Gynaecological Oncology, Barts Health NHS Trust, London E1 1FR, UK; 3 Wolfson Institute of Population Health, Barts CRUK Cancer Centre, Queen Mary University of London, Charterhouse Square, London EC1M 6BQ, UK; 4 Department of Paediatrics and Paediatric Infectious Diseases, Institute of Child’s Health, Sechenov First Moscow State Medical University (Sechenov University), 29 Shmitovskiy Proezd, 123337 Moscow, Russia; 5 Care for Long Term Conditions Division, Florence Nightingale Faculty of Nursing Midwifery and Palliative Care, King’s College London, London SE1 8WA, UK; 6 Solov’ev Research and Clinical Center for Neuropsychiatry, 43 Ulitsa Donskaya, 115419 Moscow, Russia; 7 Department of Preventive Medicine and Public Health, Universidad de Granada, 18071 Granada, Spain; 8 Department of Health Services Research and Policy, London School of Hygiene & Tropical Medicine, London WC1H 9SH, UK; 9 MRC Clinical Trials Unit, University College London, 90 High Holborn, London WC1V 6LJ, UK; 10 Department of Gynaecology, All India Institute of Medical Sciences, New Delhi 110029, India

**Keywords:** *BRCA*, risk-reducing salpingo-oophorectomy, breast cancer, meta-analysis

## Abstract

**Simple Summary:**

Women with *BRCA1* or *BRCA2* gene mutations are at increased risk of breast and ovarian cancer and often undergo operations to remove both their ovaries in order to prevent ovarian cancer. The impact of this operation on breast cancer risk is uncertain; thus, we performed a systematic review and meta-analysis to determine it. We found that this operation was not linked with a reduced risk of developing breast cancer when considering both *BRCA1* and *BRCA2* carriers together but was linked with a reduced risk of breast cancer when considering *BRCA2* carriers alone. If a woman had this operation after developing breast cancer, it was not related to a reduced chance of developing cancer in the other breast. However, it was associated with increased survival following breast cancer when considering *BRCA1* and *BRCA2* carriers together and *BRCA1* carriers alone. These findings may have important implications for counselling for women in the clinic.

**Abstract:**

Background: Risk-reducing salpingo-oophorectomy (RRSO) is the gold standard method of ovarian cancer risk reduction, but the data are conflicting regarding the impact on breast cancer (BC) outcomes. This study aimed to quantify BC risk/mortality in *BRCA1*/*BRCA2* carriers after RRSO. Methods: We conducted a systematic review (CRD42018077613) of *BRCA1*/*BRCA2* carriers undergoing RRSO, with the outcomes including primary BC (PBC), contralateral BC (CBC) and BC-specific mortality (BCSM) using a fixed-effects meta-analysis, with subgroup analyses stratified by mutation and menopause status. Results: RRSO was not associated with a significant reduction in the PBC risk (RR = 0.84, 95%CI: 0.59–1.21) or CBC risk (RR = 0.95, 95%CI: 0.65–1.39) in *BRCA1* and *BRCA2* carriers combined but was associated with reduced BC-specific mortality in BC-affected *BRCA1* and *BRCA2* carriers combined (RR = 0.26, 95%CI: 0.18–0.39). Subgroup analyses showed that RRSO was not associated with a reduction in the PBC risk (RR = 0.89, 95%CI: 0.68–1.17) or CBC risk (RR = 0.85, 95%CI: 0.59–1.24) in *BRCA1* carriers nor a reduction in the CBC risk in *BRCA2* carriers (RR = 0.35, 95%CI: 0.07–1.74) but was associated with a reduction in the PBC risk in *BRCA2* carriers (RR = 0.63, 95%CI: 0.41–0.97) and BCSM in BC-affected *BRCA1* carriers (RR = 0.46, 95%CI: 0.30–0.70). The mean NNT = 20.6 RRSOs to prevent one PBC death in *BRCA2* carriers, while 5.6 and 14.2 RRSOs may prevent one BC death in BC-affected *BRCA1* and *BRCA2* carriers combined and *BRCA1* carriers, respectively. Conclusions: RRSO was not associated with PBC or CBC risk reduction in *BRCA1* and *BRCA2* carriers combined but was associated with improved BC survival in BC-affected *BRCA1* and *BRCA2* carriers combined and *BRCA1* carriers and a reduced PBC risk in *BRCA2* carriers.

## 1. Introduction

*BRCA1* and *BRCA2* mutation carriers have a ~17–44% risk of ovarian cancer (OC) and ~69–72% risk of breast cancer (BC) [1,2,3,4]. *BRCA* carriers can benefit from lifestyle and reproductive advice incorporating breast feeding, contraception and informed reproductive decision making, including preimplantation genetic diagnosis (PGD) [5]. Risk-reducing mastectomy (RRM) [6], screening (breast MRI/mammography) and medical prevention (selective oestrogen receptor modulators) are available options used to reduce BC risk [7,8]. Primary surgical prevention in the form of risk-reducing salpingo-oophorectomy (RRSO) is the most effective option and gold standard for OC risk reduction, especially given the absence of an effective national OC screening program. It is associated with reductions in epithelial OC risk (80–95%) [9,10,11,12] and all-cause (60–76%) and OC-specific (75–95%) mortality. It is associated with minimal surgical morbidity and is usually undertaken through minimal access surgery, including laparoscopic, robotic and other novel approaches [13,14].

Various RRSO uptake rates reaching as high as 78% have been reported amongst *BRCA1*/*BRCA2* mutation carriers [9,15,16,17,18,19,20,21,22,23,24,25,26,27,28,29,30,31,32,33,34,35,36,37,38,39,40,41,42,43,44,45]. Decision making is a complex and dynamic process which changes with time and is influenced by multiple factors. Premenopausal RRSO leads to premature surgical menopause, which has detrimental long-term health sequelae (increased risk of heart disease, osteoporosis, vasomotor symptoms, sexual dysfunction, neurocognitive decline), particularly if women are unable to use hormone replacement-therapy (HRT) for reason such as a personal history of BC [46,47,48,49,50,51,52,53,54,55,56]. The impact of RRSO on BC risk is a critically important factor for women considering surgical prevention [57,58,59]. Earlier publications have suggested that RRSO is associated with a 46–62% reduction in primary BC risk, 41–59% reduction in contralateral BC risk and a 54–90% reduction in BC-specific mortality [10,11,22,28,58,60,61,62,63,64,65]. However, more recent data have led researchers to question this benefit of a reduction in BC risk [66,67,68,69]. As randomised controlled trials (RCTs) investigating the health effects of risk-reducing surgeries are unethical and unacceptable in the case of *BRCA* carriers, evaluations of efficacy are restricted to observational studies. Consequently, the risk estimates are subject to additional potential biases. Several methodological issues have been identified, which may have affected risk estimates, leading to conflicting results. These include the study design (retrospective/prospective samples; case-control/cohort studies), differing inclusion criteria, differing sample sizes and different types of selection bias (indication, cancer-induced testing, immortal person-time, familial event biases) [67,70,71].

Accurate information on the pros/cons and efficacy of risk reduction, along with the side effects and surgical risks, must form the basis of informed counselling for surgical prevention offered to *BRCA* carriers. Despite the methodological limitations of studies investigating the health effects of RRSO, the data consistently show a reduction in OC risk. However, given the existence of contradictory data, the same is not true for the impact on BC risk following RRSO. In order to aid clinicians in counselling *BRCA* carriers faced with the decision as to whether or not and when to undergo RRSO and to help patients make informed decisions, we undertook a systematic review of the available evidence regarding the association between RRSO and the risk of BC development. This is particularly important given the recent conflicting data. The aim of this review is to summarise the published evidence of BC outcomes following RRSO in *BRCA* carriers.

## 2. Materials and Methods

### 2.1. Search Strategy and Selection Criteria

In this systematic review, we used a comprehensive three-step search strategy to identify relevant studies. Using the NICE Evidence Service’s healthcare databases advanced search (HDAS) tool, first, we simultaneously searched the following seven databases from the time of inception to 14 June 2022: Pubmed, Medline, Embase, CINAHL, PsycINFO, PROSEPRO and Cochrane. A common search strategy (Supplementary Materials, Table S1) was developed for database searching via HDAS using a combination of free text and controlled vocabulary (MeSH terms). Second, reference lists of publications retrieved in the first step were screened for relevant studies. Third, we searched additional web-based platforms, including specialised journals, Google searches for grey literature, conference proceedings and clinical trial registries (ISRCTN and ClinicalTrials.gov [accessed on 14 June 2022]).

To increase the sensitivity of our search, no restriction was placed on language, geographical location, year of publication or the type of study. The search was limited to human studies and re-run prior to the final analyses in order to ensure that recently published studies were retrieved for inclusion.

The articles were independently screened by two authors (FG, AT) in two stages after all the identified references were transferred into a reference management software package (EndNote X8.2, Clarivate Analytics). The titles and abstracts were screened, followed by the retrieval and screening of the full-text articles, fulfilling the eligibility criteria described below. Inter-rater reliability was analysed using quantity (Q) and allocation (A) disagreements [72]. Disagreements were resolved by consensus or arbitration by a third reviewer (RM).

The predefined inclusion criteria were female *BRCA1* and *BRCA2* mutation carriers undergoing RRSO. The outcomes investigated were: (1) primary breast cancer (PBC), defined as the risk of invasive BC occurring in a previously unaffected individual; (2) contralateral breast cancer (CBC), defined as the risk of a second case of primary invasive BC occurring in a previously unaffected contralateral breast; and (3) BC-specific mortality (BCSM), defined as cause of death due to BC. We excluded studies that included participants who had a personal history of OC or had undergone prophylactic RRM prior to RRSO, as well as abstracts.

### 2.2. Data Extraction and Quality Assessment

Data were extracted by two reviewers (FG, AT) using a standardised, predesigned data extraction sheet in Microsoft Excel 2013. FG extracted data from the publications, and AT crosschecked the data for accuracy. Four main categories of data were extracted: methodological characteristics, study population, surgical interventions (RRSO/RRM) and reported outcome measures pertaining to PBC/CBC risk and BCSM. The data extraction sheet was piloted and refined before extraction. In cases where studies reported both adjusted and unadjusted data, both were collected. The risk of bias was assessed by the reviewers (FG, OB) using the Newcastle–Ottawa Scale (NOS) [73]. A low risk of bias was attributed to studies that scored four stars for selection, two stars for comparability and three stars for ascertainment of the outcome/exposure. A medium risk of bias was allocated to studies with two to three stars for selection, one for comparability and two for outcome/exposure ascertainment. All studies with scores of one for selection or outcome/exposure ascertainment or zero for any of the three domains were regarded as having a high risk of bias [74]. GRADE (Grading of Recommendations, Assessment, Development and Evaluations) was used to assess the overall quality of the evidence for each outcome. Each outcome was assigned a level of certainty in terms of the evidence. “Very low” was defined as the true effect, probably being markedly different from the estimated effect; “low” was defined as the true effect, which might be markedly different from the estimated effect; “moderate” was defined as the true effect, probably being close to the estimated effect; and “high” was defined as the true effect, being similar to the estimated effect.

### 2.3. Data Analysis

We tabulated the characteristics and reported outcome measures of all the studies for the qualitative synthesis of the data. No studies were excluded from the qualitative data synthesis based on the risk of bias scores. The decision to perform a meta-analysis (quantitative data synthesis) was made a posteriori to ensure that a sufficient number of studies with similar characteristics were available. Case-control studies were excluded from the quantitative synthesis due to their less robust study design, smaller number of outcome events and higher risk of bias. For quantitative synthesis, we compared the BC outcomes in *BRCA1* and *BRCA2* carriers undergoing RRSO with those of *BRCA1* and *BRCA2* carriers not undergoing RRSO. As the studies varied in their outcome measures, to ensure comparability between studies, the relative risk (RR) was calculated using raw data independently extracted by the authors, FG, OB or RM, using 2 × 2 tables. FG and OB extracted the data, and RM crosschecked the data for accuracy. The investigators were contacted for those studies in which raw data were missing from the published manuscript. In instances where two or more studies had overlapping datasets, the study with the least risk of bias or highest quality was used for pooling. In instances where the risk of bias/quality of the study was deemed to be equivalent between overlapping studies, the study with the largest number of events was included. All the analyses were performed using the package “meta” of the R Studio software (version 3.5.1). Since the studies differed in terms of the year of study, geographical location, confounders and reported measurements of the effect size (hazard ratio (HR)/odds ratio (OR)/relative risk (RR)), the relative risks and 95% confidence intervals calculated from the raw data were pooled based on a random effects model. The DerSimonian–Laird estimate was used to assess the between-study variance. To determine the extent of inter-study variation, we performed heterogeneity tests with Higgins’ I^2^ statistic to measure the proportion of the observed variance that reflected the true effect sizes [75]. An I^2^ ≥50% was considered to represent significant inter-study variation [76].

A baseline analysis was performed to examine the PBC risk, CBC risk and BCSM amongst both *BRCA1* and *BRCA2* carriers. A subgroup analysis was performed according to *BRCA* and menopause status (women aged <50 years were assumed to be premenopausal and those aged ≥50 years were assumed to be postmenopausal). It was not possible to investigate sources of model heterogeneity because of the small number of studies in each analysis.

We calculated numbers-needed-to-treat (NNT) values for all the statistically significant outcomes using the ‘treat as one trial’ approach: $\mathrm{NNT}=\frac{1}{\left(1-\mathrm{RR}\right)*\pi_{0}}$, for RR<1, and $\mathrm{NNT}=\frac{1}{\left(\mathrm{RR}-1\right)*\pi_{0}}$, for RR>1, where RR—pooled relative risk and $\pi_{0}$—risk for the control (unexposed) group. The NNT values, together with 95% confidence intervals, were calculated for the minimum, maximum and mean $\pi_{0}$ across the correspondent studies.

Our work conformed to the Preferred Reporting Items for Systematic Reviews and Meta-Analysis (PRISMA) guidelines. Details of the protocol were registered prospectively in the international PROSPERO database (registration number CRD42018077613). Our work was exempt from Institutional Review Board approval, as our review summarizes already published data.

## 3. Results

### 3.1. Study Selection and Characteristics

**Figure 1:**
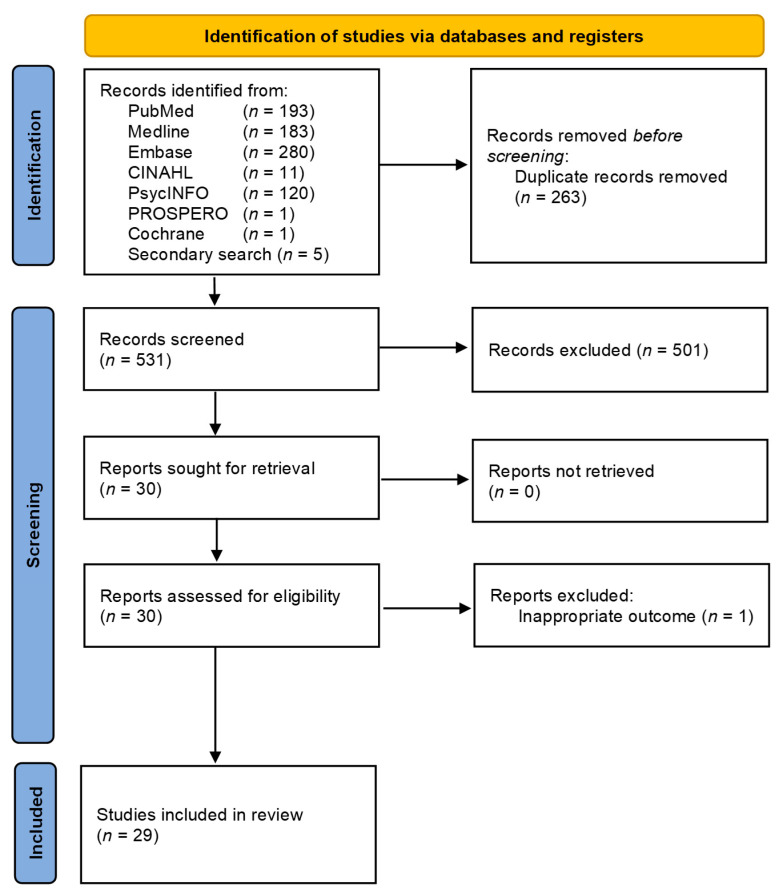
PRISMA flow diagram of study selection [77].

Searches of electronic databases and reference lists of 789 generated references (Figure 1).

On evaluation of all the titles and abstracts, 30/789 articles (3.8%) were potentially eligible for detailed assessment [9,10,11,22,24,57,58,59,60,61,62,63,64,65,66,67,68,69,78,79,80,81,82,83,84,85,86,87,88,89,90,91,92,93,94,95]. A total of 29/30 met the predefined inclusion criteria for qualitative synthesis (Table 1). One study was excluded due to the fact it reported non-BC-related outcomes.

There were high levels of agreement between the reviews (Q = 1/29, A = 2/29). Sixteen studies reported the PBC risk [10,11,22,58,60,61,62,65,67,68,79,82,93,94,95,97]. Of these studies, there were three groups of overlapping datasets: (1) the PROSE consortium datasets: Finkelman et al. 2012 [22], Kauff et al. 2008 [11], Rebbeck et al. 1999 [65], Rebbeck et al. 2002 [10] and Domchek et al. 2010 [58]; (2) the datasets of Eisen et al. 2005 [60], Kotsopoulos et al. 2012 [61] and Kotsopoulos et al. 2017 [68]; and (3) the datasets of Chang-Claude et al. 2007 [79], Mavaddat et al. 2013 [62], Mavaddat et al. 2020 [97], Heemskerk-Gerritsen et al. 2015 [67], Choi et al. 2021 [93] and Terry et al. 2018 [95]. Six studies reported the CBC risk [58,62,64,66,69,84], with Metcalfe et al. 2004 [64], Metcalfe et al. 2011 [63] and Kotsopoulos et al. 2019 [69] having overlapping datasets. Seven studies reported on BCSM [21,57,58,59,89,90,92], with Domchek et al. 2006 [57] and Domchek et al. 2010 [58] sharing the same dataset. All 29 studies were observational, with no RCT. Five studies were case-control studies, one was cross-sectional, and twenty-three were cohort in design. The size of the studies varied from 98 [82] to 8977 [61] participants, and the follow-up period ranged from 14.1 years [82] to 1.6 years [57]. The follow-up duration was not reported in seven studies [60,61,79,91,92,93]. The outcomes were routinely assessed using hospital records and self-reported questionnaires. RRM was a censoring event in twelve studies [9,11,22,58,62,67,79,82,93,94,95,97], excluded in twelve studies [10,58,60,61,62,63,64,65,66,68,69,91], included in one study [59] and not reported in four studies [83,89,90,92]. Studies investigating the PBC risk adjusted for the following confounders: age [11,65,67,68], parity [11,60,67,68,79], HRT use [11,79,97], OCP use [60,68], mutation status [67], centre [67], country of residence [68], age at menarche [68], BC family history [68] and breast feeding [68]. Studies investigating CBC adjusted for age [64], menopause [66], ascertainment [66], mutation status [64] and BC treatment (chemotherapy/radiotherapy/surgery/tamoxifen) [63,64]. Studies investigating mortality adjusted for age [57,58,59], mutation status [57,59], tumour size [59,89], nodal status [59,89], oestrogen receptor status [59,89], progesterone receptor status [89], HER2 receptor status [89], BC treatment (chemotherapy [59,89]/surgery [59]/tamoxifen [59,89]) and centre [57,58].

### 3.2. Risk of Bias

Table 2 summarises the risk of bias assessment, and Table 3 summarises the GRADE assessment for certainty of evidence per outcome.

The GRADE certainty of evidence for PBC and CBC was low, and that for BCSM was moderate. According to GRADE, all the observational studies have an initially low level of evidence. For PBC and CBC, the certainty of evidence was downgraded due to the serious risks of bias and conflicting effect sizes of the individual studies. For BCSM, the certainty of evidence was also downgraded due to serious risks of bias; however, it was upgraded when taking into account the large and consistent effect sizes of the studies.

### 3.3. Quantitative Synthesis of Results

**Figure 2:**
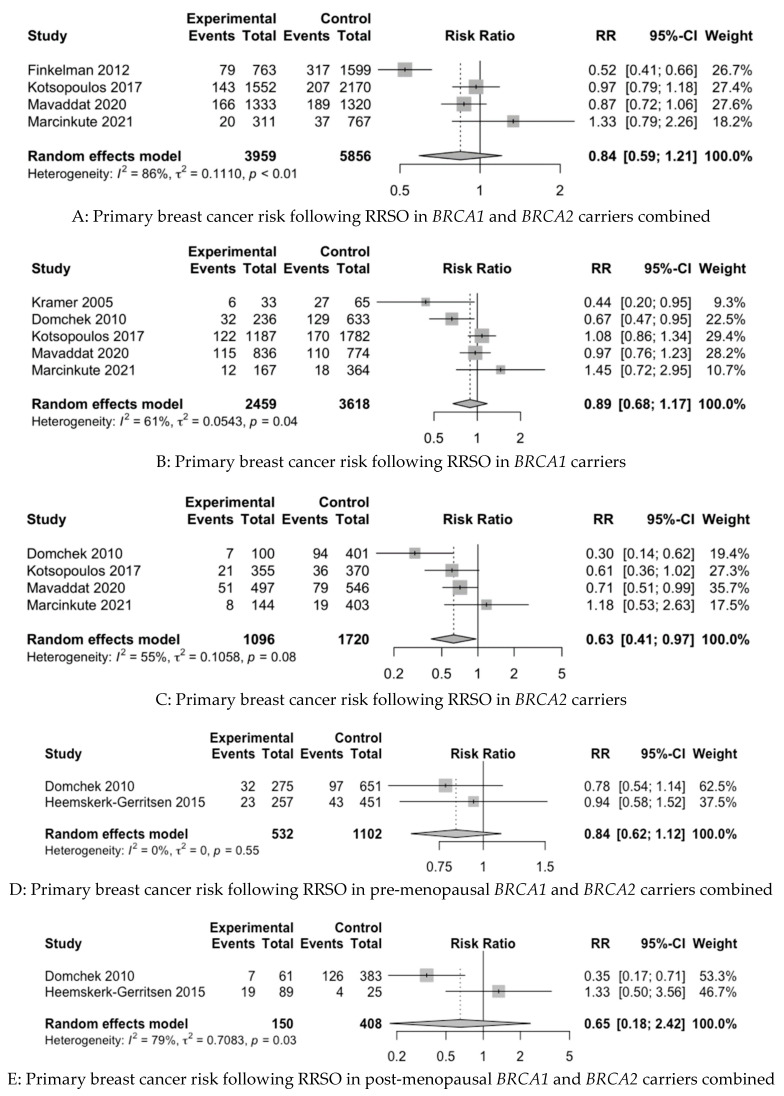

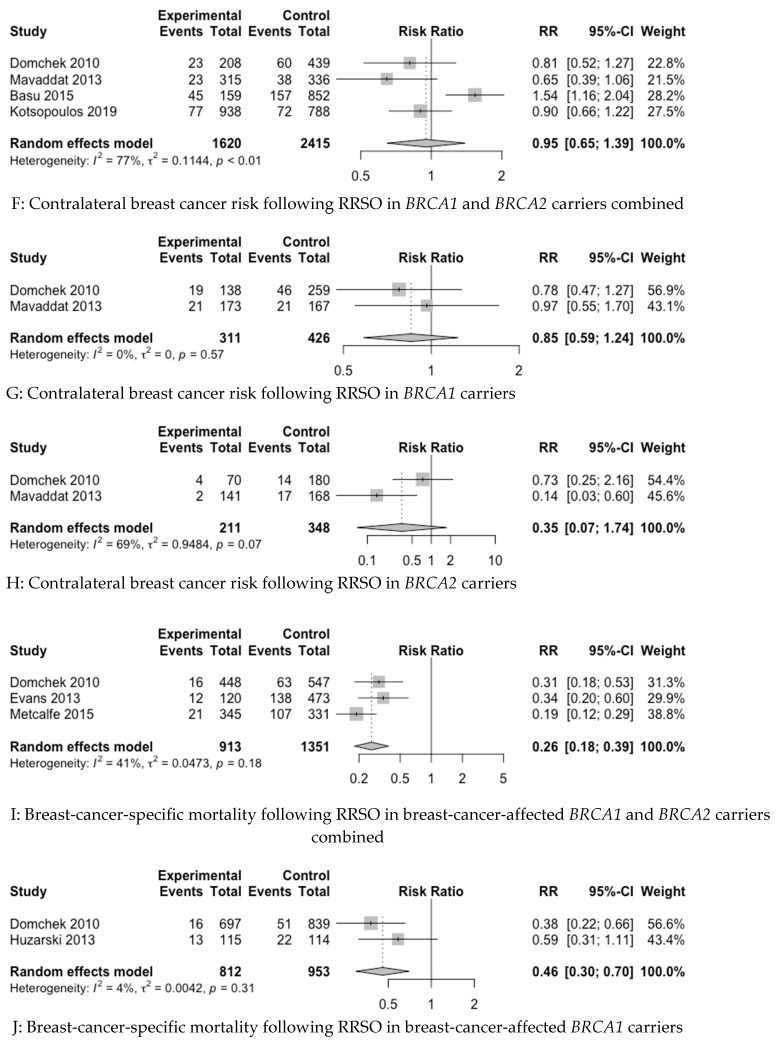
Forest plots of pooled relative risk estimates evaluating breast cancer endpoints following RRSO in *BRCA* carriers [22,58,59,62,66,67,68,69,82,89,94,97]. RRSO—risk-reducing salpingo-oophorectomy.

In the baseline meta-analysis of the PBC risk in both *BRCA1* and *BRCA2* carriers combined, four studies were included [22,68,94,97]. In the baseline meta-analysis of CBC in both *BRCA1* and *BRCA2* carriers combined, four studies were included [58,62,66,69]. It was not possible to include pooling for the PBC or CBC risk in the baseline data in the case of the studies of Kauff et al. 2002 and 2008 [9,11] and Menkiszak et al. 2016 [83], on account of the fact that both studies reported on PBC and CBC cases together, and it was not possible to differentiate between the reported cases based on the raw data. We were unable to extract raw data from two recent publications for inclusion in our meta-analysis, despite requests sent to the authors [88,90]. For BCSM in BC-affected *BRCA1* and *BRCA2* carriers combined, three studies were included [58,59,91]. Figure 2 depicts forest plots of the baseline and subgroup analyses.

Table 4 summarises the relative risks for all the baseline and subgroup analyses.

In the baseline analyses, RRSO was not statistically significantly associated with a reduction in the risk of PBC in *BRCA1* and *BRCA2* carriers combined (RR 0.84, 95%CI 0.59–1.21) or the risk of CBC in *BRCA1* and *BRCA2* carriers combined (RR 0.95, 95%CI 0.65–1.39). However, RRSO was statistically significantly associated with a reduction in BCSM in BC-affected *BRCA1* and *BRCA2* carriers combined (RR 0.26, 95%CI 0.18–0.39).

For the subgroup analysis of the PBC risk, five studies were included for BRCA1 carriers alone [58,68,82,94,97], with four studies for *BRCA2* carriers alone [58,68,94,97], two studies for pre-menopausal *BRCA1* and *BRCA2* carriers combined [58,67] and two studies for post-menopausal *BRCA1* and *BRCA2* carriers combined [58,67]. For the subgroup analysis of the CBC risk, two studies were included for *BRCA1* carriers alone [58,62], with two studies for *BRCA2* carriers alone [58,62]. Subgroup analyses stratified by menopause status for CBC were attempted; however, raw data were not available. It was possible to perform a subgroup analysis of BCSM for BC-affected *BRCA1* carriers alone [58,89], but due to lack of raw data, we were unable to do this for *BRCA2* carriers alone.

The subgroup analysis revealed that RRSO was not statistically significantly associated with a reduced PBC risk in *BRCA1* carriers alone (RR 0.89, 95%CI 0.68–1.17) or with the PBC risk in pre-menopausal (RR 0.84, 95%CI 0.62–1.12) or post-menopausal *BRCA1* and *BRCA2* carriers combined (RR 0.65, 95%CI 0.18–2.42). However, RRSO was associated with a reduced risk of PBC in *BRCA2* carriers alone (RR 0.63, 95%CI 0.41–0.97). The subgroup analysis revealed that RRSO was not associated with CBC risk reduction in *BRCA1* carriers alone (RR 0.85, 95%CI 0.59–1.24) or *BRCA2* carriers alone (RR 0.35, 95%CI 0.07–1.74). However, RRSO was associated with a reduction in the risk of BCSM in BC-affected *BRCA1* carriers in the subgroup analysis (RR 0.46, 95%CI 0.30–0.70).

The heterogeneity of the baseline and subgroup models, as measured by I^2^, ranged from 0 to 86%. The models with low heterogeneity (I^2^ < 50%) were as follows: PBC risk in premenopausal *BRCA1* and *BRCA2* carriers combined, CBC risk in *BRCA1* carriers alone, BCSM in *BRCA1* and *BRCA2* carriers combined and BCSM in *BRCA1* carriers alone. The remainder of the models had high heterogeneity (I^2^ ≥ 50%).

The mean NNT for statistically significant outcomes showed that 5.6 (4.2 to 11.8) and 14.5 (9.5 to 30.3) RRSOs are needed to prevent one death from BC in BC-affected *BRCA1* and *BRCA2* carriers combined and one death from BC in BC-affected *BRCA1* carriers alone, respectively (Table 4). Moreover, 20.6 (11.5 to 57) RRSOs are needed to prevent one PBC case in *BRCA2* carriers (Table 4).

## 4. Discussion

### 4.1. Main Findings

In this systematic review, we provide relative risk estimates of PBC risk, CBC risk and BCSM in *BRCA1* and *BRCA2* carriers following RRSO data from fourteen publications published between 2005 and 2021. The results of this meta-analysis demonstrate that RRSO was not associated with a significant reduction in the overall PBC risk or CBC risk in the analyses incorporating both *BRCA1* and *BRCA2* carriers combined, nor is CBC risk significantly reduced when analysed separately by the type of *BRCA* mutation. RRSO was associated with a significant reduction in PBC risk in *BRCA2* carriers alone, although there did not appear to be a reduction in PBC risk in *BRCA1* carriers alone.

### 4.2. Comparison with Existing Literature

It appears that, previously, we have considerably overestimated the benefit of RRSO in regard to BC risk reduction. The impact, if any, is probably restricted to *BRCA2* carriers, 70% of whom have ER-positive BC. This is in contrast with the 70% of breast cancers in *BRCA1* carriers which are triple-negative (1) and is therefore in keeping with the hypothesis that a reduction in circulating oestrogens/progesterones following RRSO would most likely reduce the risk of hormone-sensitive tumours. To some extent, this is not inconsistent with the effect seen in the case of other anti-oestrogen interventions, such as Tamoxifen, which only reduces the risk of ER-positive BC in high-risk women, or the benefit of GnRH analogues in regard to the overall survival observed in women with ER-positive BC alone [98]. Additionally, the PBC risk post-RRSO was not significantly influenced by menopausal status. There is an associated reduction in BCSM in BC-affected *BRCA1* and *BRCA2* carriers combined and BC-affected *BRCA1* carriers alone following RRSO. Although one study suggested a potential, small, non-significant reduction in BCSM in *BRCA2* carriers alone following RRSO (HR 0.87, 95%CI 0.32–2.37) [58], the effect, if true, is small, and the overall paucity of published data on BCSM in BC-affected *BRCA2* carriers precludes the ability to perform a subgroup analysis.

There are three previously published systematic reviews and meta-analyses investigating BC outcomes after RRSO in *BRCA1* and *BRCA2* carriers. Rebbeck et al., 2009 [87] investigated PBC risk over a decade ago, while more recently, Eleje et al., 2018 [99] investigated PBC risk and BCSM, and Xiao et al., 2019 [100] investigated the PBC risk, CBC risk and overall survival. The latter two analysis were published after data contradicting earlier findings on BC risk began to emerge. Nevertheless, all three of these meta-analyses concluded that, for *BRCA1* and *BRCA2* carriers combined, RRSO was associated with a statistically significant reduction in PBC risk (pooled HR 0.21, 95%CI 0.12–0.39; HR 0.64, 95%CI 0.43–0.96; HR 0.58, 95%CI 0.37–0.78 respectively). Xiao et al. also showed a statistically significant reduction in CBC risk following RRSO in *BRCA1* and *BRCA2* carriers combined (pooled HR 0.50, 95%CI 0.31–0.69). Additionally, Xiao et al. reported a statistically significant increase in overall survival in BC-affected *BRCA1* and *BRCA2* carriers combined following RRSO (pooled HR 0.33, 95%CI 0.28–0.38), and Eleje et al. showed increased BCSM in *BRCA1* and *BRCA2* carriers combined following RRSO (pooled HR 0.58, 95%CI 0.39–0.88). Whilst the improved BCSM reported by others is in keeping with the results of our meta-analysis, the significant reduction in PBC/CBC risk following RRSO in *BRCA1* and *BRCA2* carriers combined, as reported by these researchers, is contrary to our findings. However, our meta-analysis found a significant reduction in *BRCA2* PBC risk resulting from RRSO. It is also important to point out that the overall GRADE quality assessment of studies used for the PBC meta-analysis is low, as these are observational studies. There are no RCTs which address the BC risk and mortality post-RRSO in *BRCA* carriers, as randomising individuals at high risk of OC/BC to a non-surgical arm would be both unethical and unacceptable for the women. It will thus not be possible to undertake an RCT on this issue, and inferences for patient care and practice will need to be drawn from well-designed observational cohort data. The previously published meta-analyses have a number of limitations. These studies did not include all/recently published data on PBC/CBC and, importantly, included overlapping datasets within the same meta-analysis, thus overstating the effect size of the earlier published literature showing a reduction in PBC/CBC risk following RRSO. They extracted reported HRs/RRs/ORs directly from the published literature and included different measurements of effect size which are not comparable within the same meta-analysis.

It appears that the conflicting risk estimates of individual studies investigating PBC/CBC risk following RRSO may be due to the various selection biases described in Table 1. Indication bias occurs because individuals with a stronger family history of OC are more likely to undergo RRSO/RRM than individuals from families with less OC/BC family history. In order to take into account possible differences in penetrance between families of *BRCA* carriers with and without RRSO, matching women who have undergone RRSO with relatives who have not, or a subgroup analysis restricted to individuals from families with an OC family history, would be more valid [67,70,71]. Cancer-induced testing bias occurs when individuals undergo *BRCA* testing because of a diagnosis of BC; thus, BC is overrepresented amongst the tested mutation carriers in the non-RRSO group, which may result in an overestimation of the risk reduction associated with RRSO. This may be overcome by commencing follow-up from the date of *BRCA* testing among cancer-unaffected individuals [67,70,71]. Immortal person-time bias is also an important limitation. If follow-up for the non-RRSO group starts at the date of *BRCA* ascertainment, for the RRSO group, the person-years of observation (PYO) between the dates of ascertainment and RRSO (cancer free by definition) should not be excluded, and these cancer-free person-years might be added to the person-years of the non-RRSO group. This allocation will reduce the cancer risk in the non-surgery group and, subsequently, prevent an overestimation of the reduction in cancer risk after RRSO [67,70,71]. Familial event bias occurs when members of the same family are selected for inclusion in the study population and the date of prophylactic surgery of the RRSO subject is not considered in the analysis. For instance, if follow-up were to start at the date of *BRCA* testing, the diagnosed BC would be counted as an event in the analysis. This would result in an overestimation of BC risk amongst women in the non-RRSO group and, consequently, an overestimation of the BC risk reduction after RRSO. To overcome this bias, the age of the control at the time of the relative’s RRSO should be used as the starting point of follow-up if this age is greater than the age at testing [67,70,71]. In a landmark paper by Heemskerk et al., the authors accounted for these biases by ensuring that their study cohort had no history of cancer at the date of *BRCA* testing, allocating all PYO before surgery, as well as a latency period of three months after RRSO, to the non-RRSO group [67]. Thereafter, PYO were allocated to the RRSO group. The follow-up of their analysis ended with the participant’s age at first BC diagnosis, age at RRM, age at diagnosis of another cancer (including OC), age at last contact, age at death or age at the study closing date, whichever came first. BC cases diagnosed during the latency period were counted as events in the non-RRSO group [67]. To estimate the association between RRSO and BC risk, the team used a Cox model with RRSO as a time-dependent variable to obtain hazard ratios and their accompanying 95% confidence intervals, using the non-RRSO group as the reference group. The variance–covariance estimation method was used to correct for non-independence of observations in the case of women from the same family [67]. The following variables were considered as potential confounders: year of birth, mutation type, centre and parity. The team concluded that the BC risk reduction after RRSO in *BRCA* carriers may have previously been overestimated because of bias. Using a design that maximally eliminated bias, they found no evidence for a protective effect of RRSO on PBC risk (HR 1.09, 95%CI = 0.67–1.77), whereas, when the team replicated the analysis of four previous publications [11,57,58,60] using their data, they found a ~50% PBC risk reduction, as estimated previously [67]. Data gathered by Kauff et al. and the PROSE consortium [11,58] were reanalysed by the authors to take into account RRSO as a time-dependent variable, accounting for immortal person-time bias (as per Heemskerk et al.) [67,96]. Upon reanalysis of Kauff et al.’s data, the revised HR reported a non-significant decrease in PBC risk (reanalysis: HR 0.50, 95%CI 0.20–1.25; original analysis: HR 0.53, 95%CI 0.29–0.96), having accounted for immortal person-time bias (the original analysis already accounted for the other aforementioned selection biases) [67,96]. Although the reanalysed PROSE data accounted for immortal person-time bias, the cancer-induced testing bias remained, and the revised HR continued to show a significant decrease in PBC risk (reanalysis: HR 0.59, 95%CI 0.42–0.82; original analysis: HR 0.51, 95%CI 0.36–0.70) [67,96].

Our meta-analysis corroborates more recent data [58,67,68,69,97] which show that RRSO does not reduce the PBC/CBC risk in either *BRCA1* or *BRCA2* carriers, contrary to the findings of earlier publications, which showed a reduction in the PBC/CBC risk post-RRSO in *BRCA1* carriers [58,60,62,65,82] and *BRCA2* carriers [11,58,62]. The subgroup analysis of CBC by mutation status is consistent with this finding, as the confidence intervals of the relative risk estimates includes ‘1’. Our finding of the associated reduction in PBC risk in *BRCA2* carriers is in keeping with a recent publication by Mavaddat et al. 2020 [97], which reported a reduction in PBC risk after >5 years (but not ≤5 years) following RRSO in *BRCA2* carriers (HR 0.51, 95%CI 0.26–0.99). However, it is important to remain cautious in the interpretation of this pooled estimate and findings regarding *BRCA2* from Mavaddat et al. 2020 [97] due to the smaller number of events and wide confidence intervals observed in the *BRCA2* cases compared to *BRCA1*. This is likely due to the smaller sample size of the *BRCA2* group. Nevertheless, more prospective data are needed to improve the precision of PBC estimates. Subgroup analysis also revealed no significant reduction in PBC after RRSO in either premenopausal or postmenopausal women. This finding is in agreement with Mavaddat et al., who did not find a statistically significant reduction in PBC risk in pre- or postmenopausal *BRCA1* and *BRCA2* carriers (*BRCA1*: premenopausal HR 1.11 (95%CI 0.82–1.50), postmenopausal HR 1.69 (95%CI 0.73–3.91); *BRCA2*: premenopausal HR 0.69 (95%CI 0.44–1.08), postmenopausal HR 1.46 (95%CI 0.66–3.19)); Chang-Claude et al. [79], who did not find a statistically significant reduction in PBC risk post-RRSO in postmenopausal *BRCA1* and *BRCA2* carriers (HR 0.5, 95%CI 0.24–1.04; HR 0.39, 95%CI 0.06–2.38 respectively); and Rebbeck et al. [10], who found no risk reduction in postmenopausal *BRCA1* and *BRCA2* carriers combined (HR 0.52, 95%CI 0.10–2.70). However, these findings are contrary to Chang-Claude et al. [79], who found a significant reduction in PBC risk in premenopausal *BRCA1* (but not *BRCA2*) carriers who underwent RRSO at < 35 years of age (HR 0.05, 95%CI 0.01–0.49), and Rebbeck et al. [10], who demonstrated a statistically significant risk reduction among premenopausal *BRCA1* and *BRCA2* carriers combined, aged 35–50 years (HR 0.49, 95%CI 0.26–0.90). In addition, Kotsopoulos et al. [61] showed a statistically significant risk reduction among postmenopausal *BRCA1* and *BRCA2* carriers combined (OR 0.13, 95%CI 0.05–0.54). It is possible that the aforementioned selection biases may have contributed to the conflicting results of the individual studies. However, it must be noted that it was not possible to extract data on the precise ages of patients grouped as pre-menopausal (<50 years) and post-menopausal (>50 years) from the published data included in our meta-analysis. Additionally, while the age of 50 is indicative, it may not be a true representation of the time of menopause, as some women may experience menopause earlier or later. It is therefore possible that there may be difference in breast cancer risk reduction depending on whether oophorectomy was performed in the peri-menopausal period, near the time of menopause or much earlier, in the case of pre-menopausal women, where oophorectomy would result in a significantly greater reduction in the number of lifetime ovulatory cycles. Therefore, our subgroup analysis of PBC risk and menopause status must be interpreted within this context.

The clinical management of cancer risk in *BRCA* carriers is complex and must consider patient preferences. RRSO remains the gold standard for preventing OC in *BRCA* carriers. Whether or not and when to undergo RRSO can be a complicated decision for many patients and may evolve over time [34]. Patient preferences need to be informed (and can be influenced) by accurate knowledge of the risks and benefits of the interventions considered. The previously perceived beneficial impact on BC risk is an important issue that has been routinely discussed during counselling and one of the key considerations in patient decision making. Our data suggest that *BRCA* carriers considering RRSO should now be counselled about the lack of consistent evidence for a reduction in BC risk. This is particularly the case for *BRCA1* carriers. It is possible that this may influence more women to consider early risk-reducing salpingectomy and delayed oophorectomy, instead, as an emerging alternative preventive strategy (although this is still only recommended in clinical trials) [101,102]. However, one case of BC can be prevented for every 20.6 unaffected *BRCA2* women undergoing RRSO. Despite the lack of benefit in reducing BC incidence, it is interesting that the results show a benefit for BCSM, with one BC-related death prevented for every 5.6 and 14.5 RRSOs performed on BC-affected *BRCA1* and *BRCA2* carriers combined and BC-affected *BRCA1* carriers alone, respectively. Among the studies included in this meta-analysis, the individual analyses reported hazard ratios showing that BCSM is reduced in BC-affected *BRCA1* carriers (HR 0.27, 95%CI 0.12–0.58 [58]; HR 0.38, 95%CI 0.19–0.77 [59]; HR 0.30 95%CI 0.12–0.75 [89]) but not in BC-affected *BRCA2* carriers (HR 0.87, 95%CI 0.32–2.2.37 [58]; HR 0.57 95%CI 0.23–1.43 [59]). In addition, in a paper reporting on BCSM in BC-unaffected *BRCA1* carriers, RRSO appeared to improve the rate of survival (HR 0.30 95%CI 0.06–1.53 [58]). The authors were unable to evaluate the impact on BC-unaffected *BRCA2* carriers, as there were no events in this group. It is difficult to explain why BCSM mortality is improved by RRSO in *BRCA1* while BC incidence is not. Clinicians must remain cautious in their interpretation of these findings. The observational studies evaluating BCSM outcomes are also affected by the aforementioned methodological biases (Table 1). Nevertheless, for a BC-affected woman, the current evidence suggests a potential greater benefit of RRSO compared to an early-salpingectomy-based approach. This, of course, needs to be balanced against the detrimental health impact of premature surgical menopause, which may result from oophorectomy in women who remain premenopausal following BC treatment, particularly given the inability of most of these women to take HRT.

### 4.3. Strengths and Limitations

Our work conformed to the PRISMA guidelines, and the protocols were prospectively registered in the PROSPERO database. The strengths of our systematic review and meta-analysis include a comprehensive search strategy, identifying all the relevant literature for inclusion, and methodologically rigorous pooled relative risk estimates of BC outcomes from published/requested raw data, resulting in standardised measures of the effect sizes of the included studies. Overlapping datasets with a greater risk of bias were excluded, ensuring that no particular dataset was overrepresented in our analyses. To limit the influence of the risk of reporting bias in our findings, all the published studies on RRSO in *BRCA* carriers were included.

Due to the lack of published raw data and inconsistency of the outcomes reported, the subgroup analysis was restricted, and it was not possible to fully investigate the effects of *BRCA1* and *BRCA2* pathogenic variants independently and the effect of menopause status on all the reported BC outcomes. This limitation, in part, could be addressed in future research through a meta-analysis based on individual patient data. Unwarranted variation in the reporting of outcomes and outcome measures between studies has been highlighted as a major limitation within women’s health research. This is being addressed by the CoRe Outcomes in Women’s and Newborn health (CROWN) initiative, which advocates for the development of a core outcome set (COS) for every woman’s health, disease and procedure. There was a large degree of statistical heterogeneity (I^2^ ≥ 50%); thus, a random effects meta-analysis was performed, which produced more conservative confidence intervals. This only partly removes the effects of heterogeneity. Another limitation is that the geographical location of the included studies was limited to Europe/North America/Israel. It is therefore possible that these results may not be generalizable to non-Caucasian populations.

### 4.4. Implications

Our findings are important and can be useful for clinical care and decision making. They can be helpful for clinicians in regard to counselling and for women in regard to decision making while factoring in the impact on breast cancer risk and survival and considering decisions in relation to risk-reducing surgery for ovarian cancer prevention. Women need to consider a number of additional factors when making this decision, including age, menopause, impact on sexual function, cardiovascular, neurological and bone health, the potential need for and ability to take HRT and the level of cancer risk reduction [56,103]. Although oophorectomy does appear to increase BC-specific survival, there are effective alternative treatments available for improving BC outcomes. RRM reduces BCSM (HR = 0.06, 95%CI = 0.01–0.46) and overall mortality (HR = 0.40, 95%CI = 0.20–0.90) in *BRCA1* carriers [104]. However, there is no significant effect on overall mortality in *BRCA2* carriers (HR 0.45, 95%CI 0.15–1.36), while the effect on BCSM is unclear due to a lack of events [104]. Tamoxifen (RR = 0.69, 95%CI 0.59–0.84), Raloxifene (RR = 0.44, 95%CI 0.24–0.80) and aromatase inhibitors (RR = 0.45, 95%CI 0.26–0.70) are statistically significantly associated with a lower PBC risk after 3–5 years of use [8,105]. Adjuvant GnRH use for ER-positive BC increases progression-free survival, recurrence-free survival and overall survival [106]. It is clear that larger, prospective, well-designed studies which minimise the earlier methodological biases are needed to further improve the power and precision of risk estimates of the impacts on BC risk and BCSM following RRSO in *BRCA* carriers. This is necessary for women’s ability to make better-informed decisions in the future. Clinicians should emphasise that the decision to undergo salpingo-oophorectomy, in the case of both BC-unaffected and -0affected *BRCA* women, must primarily be for OC prevention, and that there are well-established alternative strategies available to reduce BC risk and prolong survival.

## 5. Conclusions

This systematic review and meta-analysis of fourteen publications found that RRSO is not associated with a significant reduction in the overall PBC risk or CBC risk in *BRCA1* and *BRCA2* carriers combined or in *BRCA1* carriers alone but is associated with a significant reduction in PBC risk in *BRCA2* carriers alone. RRSO is not associated with a reduction in CBC risk for either BRCA mutation type alone. Furthermore, RRSO is associated with improved BC survival in BC-affected *BRCA1* and *BRCA2* carriers combined and in *BRCA1* carriers alone.

This is the most comprehensive review of this topic to date, and it indicates that, previously, the benefits of RRSO for breast cancer outcomes may have been considerably overestimated. *BRCA* carriers considering RRSO should be counselled about the lack of consistent evidence for a reduction in BC risk.

## Figures and Tables

**Table 1:** Qualitative data synthesis of studies reporting breast cancer outcomes following oophorectomy in *BRCA* carriers.

Studies	Country	Study Design	Population	Intervention	RRM	Outcomes	Reported Outcome Measures (CI 95%)	Follow-Up	Potential Selection Bias
Chang-Claude 2007 [79]	Europe and Canada	Multicentre prospective cohort, retrospective analysis (EMBRACE/HEBON/ GENEPSO/IBCCS datasets)	BC-affected and -unaffected *BRCA1*/*BRCA2* carriers (n = 1601)	RRSO	Censored	PBC risk	* HR 0.57 (0.29–1.09)	Not reported	Immortal person-time Familial event
Choi 2021 [93]	US, Australia, Canada	Multicentre prospective cohort (BCFR dataset)	Unaffected *BRCA1*/*BRCA2* carriers (n = 4575)	RRSO	Censored	PBC risk	*BRCA1*: HR 0.28 (0.10–0.63); *BRCA2* HR 0.19 (0.06–0.71)	Not reported	Familial event
Eisen 2005 [60]	Europe, USA, Israel	Multicentre matched case-control	BC-affected (cases: 2283) and -unaffected (controls: 2286) *BRCA1*/*BRCA2* carriers (n = 4569; 1439 matched sets)	RRSO	Excluded	PBC risk	** OR 0.46 (0.32–0.65) * OR 0.46 (0.32–0.65)	Not reported	Cancer-induced testing
Finkelman 2012 [22]	Europe and USA	Multicentre prospective cohort (PROSE consortium)	Unaffected *BRCA1*/*BRCA2* carriers (n = 4649)	RRSO	Censored	PBC risk	HR 0.62 (0.47–0.83)	Mean 6.5 years for RRSO group from ascertainment; 4.5 years for non-RRSO group from ascertainment	Immortal person-time Indication
Heemskerk-Gerritsen 2015 [96]	Netherlands	Multicentre cohort (both retrospective and prospective—HEBON dataset)	Unaffected *BRCA1*/*BRCA2* carriers (n = 822)	RRSO	Censored	PBC risk	* HR 1.09 (0.67–1.77)	Mean 6.8 years (range 0.5–17.4) for RRSO group; 3.1 years (0.1–15.9) for non-RRSO group from ascertainment	-
Kauff 2008 [11]	Europe and USA	Multicentre prospective cohort (PROSE consortium)	Unaffected *BRCA1*/*BRCA2* carriers (n = 597)	RRSO	Censored	PBC risk	HR 0.53 (0.29–0.96)	Mean 3.03 years for RRSO group from date of surgery; 2.77 years for non-RRSO group from ascertainment	Immortal person-time
Kotsopoulos 2012 [61]	12 countries	Multicentre matched case-control	BC-affected (cases-3914) and -unaffected (controls-5063) *BRCA1*/*BRCA2* carriers (n = 8977; 2854 matched sets)	RRSO	Excluded	PBC risk	** OR 0.52 (0.40–0.66)	Not reported	Familial event Immortal person-time Cancer-induced testing
Kotsopoulos 2017 [68]	12 countries	Multicentre prospective cohort	Unaffected *BRCA1*/*BRCA2* carriers from ascertainment (n = 3722)	RRSO	Excluded	PBC risk	* HR 0.89 (0.69–1.14)	Mean 5.6 years (range 0–21.2)	Indication Immortal person-time
Kramer 2005 [82]	USA	Prospective cohort	Unaffected *BRCA1* carriers (n = 98)	RRSO	Censored	PBC risk	** HR 0.38 (0.15–0.97)	Mean 14.1 years from surgery	Indication
Marcinkute 2021 [94]	UK	Prospective cohort	Unaffected *BRCA1*/*BRCA2* carriers (n = 887)	RRSO	Censored	PBC risk	HR = 0.77 (0.45–1.34)	Mean 6.26 years from ascertainment	Indication
Mavaddat 2020 [97]	Europe, US, Australia, New Zealand	Multicentre prospective cohort (EMBRACE/HEBON/ GENEPSO/ IBCCS/ kConFab/BCFR/MUV/ INHERIT/OUH/GC-HBOC/NIO/CNIO/HCSC/LUND-BRCA/ STOCKHOLM-BRCA/IHCC/ MODSQUAD datasets)	Unaffected *BRCA1*/*BRCA2* carriers (n = 3877)	RRSO	Censored	PBC risk	*BRCA1*: HR 1.23 (0.94–1.61) *BRCA2*: HR 0.88 (0.62–1.24)	*BRCA1*: mean 5.60 (SD 3.67) person-years *BRCA2*: mean 5.03 (SD 3.44) person-years	-
Mavaddat 2013 [62]	UK, Ireland	Multicentre prospective cohort (EMBRACE)	Unaffected *BRCA1*/*BRCA2* carriers (n = 988)	RRSO	Censored	PBC risk; CBC risk	* HR 0.62 (0.35–1.09) * HR 0.59 (0.35–0.99)	Mean 3 years	Indication
Rebbeck 1999 [65]	USA	Multicentre retrospective matched case-control	Unaffected *BRCA1* carriers (n = 122; cases 43; controls 79)	RRSO	Excluded	PBC risk	** HR 0.53 (0.33–0.84)	Cases: mean 9.6 years (range <1–36) from surgery; controls: mean 8.1 years (range <1–43) after the time of the matched case’s surgery	Indication
Rebbeck 2002 [10]	Europe, USA	Multicentre retrospective matched case-control	Unaffected *BRCA1*/*BRCA2* carriers (n = 241; 99 cases; 142 controls)	RRSO	Excluded	PBC risk	** HR 0.47 (0.29–0.77)	Cases: mean 10.7 years (range 0.17–42.8) after surgery; controls: 11.9 years (0.34–42.5) after time of matched case’s surgery	Indication
Terry, 2018 [95]	US, Australia, Canada	Multicentre prospective cohort (BCFR, kConFab datasets)	Unaffected *BRCA1*/*BRCA2* carriers (n = 1289)	RRSO	Censored	PBC risk	* HR 1.04, (0.87–1.24)	Mean 10.7 years from ascertainment	Familial event
Basu 2015 [66]	UK	Cross sectional (both prospective and retrospective)	BC-affected *BRCA1*/*BRCA2* carriers (n = 782 univariable analysis; n = 450 multivariable analysis)	RRSO	Excluded	CBC risk	* HR 0.72 (0.36–1.41) ** HR 0.83 (0.46–1.50)	Median 7.8 years (range 0–37 years) from first BC	Indication
Kotsopoulos 2019 [69]	Canada, USA, Europe	Multicentre prospective cohort	BC-affected *BRCA1*/*BRCA2* carriers (n = 2303)	RRSO	Excluded	CBC risk	* HR 0.92 (0.68–1.25)	Mean 9.8 years	Indication
Metcalfe 2004 [64]	Canada, USA	Multicentre retrospective cohort	BC-affected *BRCA1*/*BRCA2* carriers or mutation status unknown but from a family with known *BRCA1*/*BRCA2* mutation (n = 336)	RRSO	Excluded	CBC risk	* HR 0.41 (0.18–0.90)	Mean 9.2 years from first BC	Indication Familial event
Metcalfe 2011 [84]	Canada, USA	Multicentre retrospective cohort	BC-affected *BRCA1*/*BRCA2* carriers or mutation status unknown but from a family with known *BRCA1*/*BRCA2* mutation (n = 810)	RRSO	Excluded	CBC risk	* RR 0.48 (0.27–0.82) ** RR 0.49 (0.32–0.77)	Mean 11.1 years (range 0.1–32.9) from first BC	-
Kauff 2008 [11]	Europe, USA	Multicentre prospective cohort (PROSE consortium)	BC-affected and -unaffected *BRCA1*/*BRCA2* carriers (n = 597)	RRSO	Censored	~BC risk	** HR 0.32 (0.08–1.20)	Mean 2.9 years	Immortal person-time
Kauff 2002 [9]	USA	Single-centre prospective cohort	BC-affected and -unaffected *BRCA1*/*BRCA2* carriers (n = 170)	RRSO	Censored	~BC risk	** HR 0.32 (0.08–1.20)	127 women-years in RRSO group from surgery, and 120 women-years in non-RRSO group from ascertainment	Immortal person-time
Menkiszak 2016 [83]	Poland	Single-centre cohort	BC-affected and -unaffected *BRCA1* carriers (n = 195)	RRSO	Not reported	~BC risk	4.63% incidence of BC post RRSO	Mean 6.7 years	Indication Familial event Immortal person-time Cancer-induced testing
Brekelmans 2005 [90]	Netherlands	Single-centre retrospective case-control	BC-affected *BRCA1* carriers (cases = 223); sporadic BC cases in women at population-level risk (controls=446)	RRSO	Not reported	BC-specific mortality	HR 0.38 (0.10–2.07)	Median 5.1 years	Cancer-induced testing Immortal person-time Familial event
Evans 2013 [91]	UK	Single-centre retrospective cohort	BC-affected *BRCA1*/*BRCA2* carriers (n = 718)	RRSO	Excluded	BC-specific mortality	^#^ HR 0.46 (0.27–0.78)	Not reported	-
Metcalfe 2015 [59]	Canada, USA	Multicentre retrospective cohort	BC-affected *BRCA1*/*BRCA2* carriers or mutation status unknown but from a family with known *BRCA1*/*BRCA2* mutation (n = 676)	RRSO	Included	BC-specific mortality	* HR 0.46 (0.27–0.79) ** HR 0.47 (0.29–0.76)	Mean 12.5 years (0.7–20.0) from first BC	Indication Cancer-induced testing
Domchek 2006 [57]	Europe, USA	Multicentre prospective cohort (PROSE consortium)	Unaffected *BRCA1*/*BRCA2* carriers (n = 426 primary analysis)	RRSO	Excluded	BC-specific mortality	* HR 0.10 (0.02–0.71)	Mean 3.6 years (SD 3.7) for RRSO group and mean 1.6 years (SD 1.2) for no RRSO group from BC to death	Cancer-induced testing
Domchek 2010 [58]	Europe, USA	Multicentre prospective cohort (PROSE consortium)	BC-affected and -unaffected *BRCA1*/*BRCA2* carriers (n = 2482)	RRSO	Censored	PBC risk; CBC risk; BC-specific mortality	PBC: * HR 0.54 (0.37–0.79); CBC * HR 1.00 (0.56–1.77); mortality: * HR 0.40 (0.26–0.61)	Median 3.65 years (range 0.52–27.4) for RRSO group from surgery; 4.29 years (range 0.5–27.9) for non-RRSO group from ascertainment	Cancer-induced testing Immortal person-time
Huzarski, 2013 [89]	Poland	Multicentre prospective cohort (Polish Hereditary Breast Cancer Consortium)	BC-affected *BRCA1* Polish founder mutation carriers and non-carriers (n = 3345)	RRSO	Not reported	BC-specific mortality	* HR 0.30 (0.12–0.75) ** HR 0.31 (0.13–0.77)	Not reported	Indication
van Sprundel 2005 [92]	Netherlands	Multicentre retrospective cohort	BC-affected *BRCA1*/*BRCA2* carriers (n = 148)	RRSO	Not reported	BC-specific mortality	* HR 0.28 (0.07–1.11) ** HR 0.15 (0.04–0.51)	Not reported	Familial event bias

PBC—primary breast cancer; CBC—contralateral breast cancer; BC—breast cancer; RRSO—risk-reducing salpingo-oophorectomy; EMBRACE—epidemiological study of familial breast cancer; HEBON—hereditary breast and ovarian cancer study, the Netherlands; IBCCS—international *BRCA1/2* carrier cohort study; PROSE—prevention and observation of surgical endpoints; kConFab—Kathleen Cuningham Foundation Consortium for Research Into Familial Breast Cancer Follow-Up Study; BCFR—Breast Cancer Family Registry; GENEPSO—Gene Etude Prospective Sein Ovaire; MUV—Medical University Vienna; GC-HBOC—German Consortium for Hereditary Breast and Ovarian Cancer; CNIO—Centro Nacional de Investigaciones Oncologicas; IHCC—International Hereditary Cancer Centre; MODSQUAD—Modifier Study of Quantitative Effects on Disease. * adjusted; ** unadjusted. ~ PBC and CBC. ^#^ overall survival following RRSO in BC-affected women.

**Table 2:** Risk of bias assessment using the Newcastle–Ottawa Score.

Studies	Selection	Comparability	Outcome/Exposure
Primary Breast Cancer risk			
Chang-Claude 2007 [79]	Medium (***)	Medium (*)	High (*)
Choi 2021 [93]	Low (****)	Low (**)	Medium (**)
Domchek 2010 [58]	Medium	Medium (*)	Medium (**)
Eisen 2005 [60]	High (*)	High (-)	High (*)
Finkelman 2012 [22]	Medium (***)	Medium (*)	Low (***)
Heemskerk-Gerritsen 2015 [96]	Low (****)	Low (**)	Low (***)
Kauff 2008 [11]	High (*)	High (-)	High (*)
Kotsopoulos 2012 [61]	High (*)	High (-)	High (*)
Kotsopoulos 2017 [68]	Medium (***)	Medium (*)	Medium (**)
Kramer 2005 [82]	Medium (***)	Medium (*)	Medium (**)
Marcinkute, 2021 [94]	Low (****)	Low (**)	Medium (**)
Mavaddat 2020 [97]	Low (****)	Low (**)	Low (***)
Mavaddat 2013 [62]	Low (****)	Medium (*)	Medium (**)
Rebbeck 1999 [65]	Medium (***)	High (-)	Medium (**)
Rebbeck 2002 [10]	Medium (***)	High (-)	Medium (**)
Terry 2018 [95]	Medium (***)	Medium (*)	Medium (**)
Contralateral Breast Cancer risk			
Basu 2015 [66]	Low (****)	Medium (*)	Low (***)
Domchek 2010 [58]	Medium	Medium (*)	Medium (**)
Kotsopoulos 2019 [69]	Low (****)	Medium (*)	Low (***)
Mavaddat 2013 [62]	Low (****)	Medium (*)	Medium (**)
Metcalfe 2004 [64]	Medium (***)	High (-)	Medium (**)
Breast Cancer Risk^+^			
Kauff 2008 [11]	High (*)	Medium (*)	Medium (**)
Kauff 2002 [9]	High (*)	Medium (*)	Medium (**)
Menkiszak 2016 [83]	High (*)	High (-)	High (*)
Breast-Cancer-Specific Mortality			
Brekelmans 2005 [90]	Medium	High (-)	High (*)
Evans 2013 [91]	Low (****)	Low (**)	Medium (**)
Metcalfe 2015 [59]	Low (****)	Medium (*)	Medium (**)
Domchek 2006 [57]	Medium	Medium (*)	Medium (**)
Domchek 2010 [58]	Medium	Medium (*)	Medium (**)
Huzarski, 2013 [89]	Medium	Medium (*)	Medium (**)
van Sprundel 2005 [92]	Medium	Medium (*)	Medium (**)

Low risk of bias: studies that scored four stars for selection, two stars for comparability and three stars for ascertainment of the outcome/exposure. Medium risk of bias: studies that scored two to three stars for selection, one for comparability and two for outcome/exposure ascertainment. High risk of bias: studies that scored one or zero for selection, zero for comparability and one or zero for outcome/exposure ascertainment. ^+^ Primary and contralateral breast cancer risk.

**Table 3:** Grading of Recommendations, Assessment, Development and Evaluations (GRADE) assessment of certainty of evidence per outcome.

Outcome	Number of Studies	Certainty of Evidence (GRADE)
Primary breast cancer	16	Low *
Contralateral breast cancer	6	Low *
Breast-cancer-specific mortality	7	Moderate **

* All included studies were observational studies, which had an initially low level of evidence. Certainty of evidence was downgraded, since there were serious risks of bias and conflicting size effects. ** All included studies were observational studies, which had an initially low level of evidence. Certainty of evidence was downgraded due to serious risks of bias. However, certainty of evidence was upgraded when taking into account the large and consistent size effect.

**Table 4:** Summary of pooled relative risks, confidence intervals and numbers needed to treat.

Breast Cancer Risk/Mortality after Risk-Reducing Salpingo-Oophorectomy	Relative Risk (95%CI)	Min NNT (95%CI)	Mean NNT (95%CI)	Max NNT (95%CI)
PBC risk in *BRCA1* and *BRCA2* carriers combined	0.84 (0.59–1.21)	-	-	-
PBC risk in *BRCA1* carriers	0.89 (0.68–1.17)	-	-	-
PBC risk in *BRCA2* carriers	0.63 (0.41–0.97)	57.05 (35.88–607.28)	20.55 (12.93–218.75)	11.47 (7.22–122.1)
PBC risk in pre-menopausal *BRCA1* and *BRCA2* carriers combined	0.84 (0.62–1.12)	-	-	-
PBC risk in post-menopausal *BRCA1* and *BRCA2* carriers combined	0.65 (0.18–2.42)	-	-	-
CBC risk in *BRCA1* and *BRCA2* carriers combined	0.95 (0.65–1.39)	-	-	-
CBC risk in *BRCA1* carriers	0.85 (0.59–1.24)	-	-	-
CBC risk in *BRCA2* carriers	0.35 (0.07–1.74)	-	-	-
Mortality in BC-affected *BRCA1* and *BRCA2* carriers combined	0.26 (0.18–0.39)	11.79 (10.58–14.18)	5.58 (5–6.71)	4.2 (3.77–5.05)
Mortality in BC affected *BRCA1* carriers	0.46 (0.30–0.70)	30.29 (23.44–54.81)	14.51 (11.23–26.26)	9.54 (7.38–17.27)
PBC risk in *BRCA1* and *BRCA2* carriers combined	0.84 (0.59–1.21)	-	-	-

PBC—primary breast cancer, CBC—contralateral breast cancer, BC—breast cancer, NNT—numbers needed to treat.

## Data Availability

Data are contained within the article or Supplementary Materials.

## Supplementary Materials

The following supporting information can be downloaded at: https://www.mdpi.com/article/10.3390/cancers15051625/s1, Table S1: Search strategy for literature search.
