# Supplementary material for: Breast Cancer Risk and Breast-Cancer-Specific Mortality following Risk-Reducing Salpingo-Oophorectomy in BRCA Carriers: A Systematic Review and Meta-Analysis
Source: Cancers (Basel). 2023 Mar 6;15(5):1625. doi: 10.3390/cancers15051625 (PMC10001253; doi:10.3390/cancers15051625)
Supplement: Supplementary file 1 [file cancers-15-01625-s001.zip › cancers-2186407-supplementary.pdf]

cancers MDPI

Table S1. Search strategy for literature search.

**Eligibility criteria**	Participants: BRCA1/BRCA2 mutation carriers<br>Intervention: RRSO<br>Comparison: No RRSO<br>Outcomes: PBC risk, CBC risk, BC specific mortality
**Data sources**	MEDLINE (1946 to 2022), EMBASE (1974 to 2022), Pubmed (1996 to 2022), CINAHL (1937 to 2022), PsychINFO (1806 to 2022), PROSPERO (2011 to 2022), Cochrane (1999 to 2022), citation searching, specialist journals, grey literature (conference proceedings/Google search), clinical trial registries (ISRCTN registry/ClinicalTrials.gov registry)
**Search strategy for database searching**	
1.	(BRCA).ti,ab
2.	exp "BRCA"/
3.	(BRCA AND "1 OR 2").ti,ab
4.	exp "BRCA AND 1 OR 2"/
5.	(BRCA AND 1).ti,ab
6.	exp "BRCA AND 1"/
7.	(BRCA AND 2).ti,ab
8.	1 OR 2 OR 3 OR 4 OR 5 OR 6 OR 7 OR 8
9.	(OOPHORECTOMY).ti,ab
10.	exp "OOPHERECTOMY"/
11.	(OVARIECTOMY).ti,ab
12.	exp "OVARIECTOMY"/
13.	(SALPINGECTOMY).ti,ab
14.	exp "SALPINGECTOMY"/
15.	(OOPHORECTOMY).ti,ab
16.	exp "OOPHORECTOMY"/
17.	(OVARIECTOMY).ti,ab
18.	exp "OVARIECTOMY"/
19.	(SALPINGECTOMY).ti,ab
20.	exp "SALPINGECTOMY"/
21.	(SALPINGO AND OOPHORECTOMY).ti,ab
22.	exp "SALPINGO AND OOPHORECTOMY"/
23.	9 OR 10 OR 11 OR 12 OR 13 OR 14 OR 15 OR 16 OR 17 OR 18 OR 19 OR 20 OR 21 OR 22
24.	(BREAST NEOPLASMS).ti,ab
25.	exp "BREAST NEOPLASMS"/
26.	(BREAST CANCER).ti,ab
27.	exp "BREAST CANCER"/
28.	24 OR 25 OR 26 OR 27
29.	8 AND 23 AND 28

RRSO-risk reducing salpingo-oophorectomy, PBC-primary breast cancer, CBC- contralateral breast cancer, BC-breast cancer.
